# The Australian Biosecurity Genomic Database: a new resource for high-throughput sequencing analysis based on the National Notifiable Disease List of Terrestrial Animals

**DOI:** 10.1093/database/baae084

**Published:** 2024-08-28

**Authors:** Jana Batovska, Natasha D Brohier, Peter T Mee, Fiona E Constable, Brendan C Rodoni, Stacey E Lynch

**Affiliations:** Agriculture Victoria Research, AgriBio Centre for AgriBioscience, 5 Ring Road, Bundoora, Victoria 3083, Australia; Agriculture Victoria Research, AgriBio Centre for AgriBioscience, 5 Ring Road, Bundoora, Victoria 3083, Australia; Agriculture Victoria Research, AgriBio Centre for AgriBioscience, 5 Ring Road, Bundoora, Victoria 3083, Australia; School of Applied Systems Biology (SASB), La Trobe University, Bundoora, Melbourne, Victoria 3086, Australia; Agriculture Victoria Research, AgriBio Centre for AgriBioscience, 5 Ring Road, Bundoora, Victoria 3083, Australia; School of Applied Systems Biology (SASB), La Trobe University, Bundoora, Melbourne, Victoria 3086, Australia; Agriculture Victoria Research, AgriBio Centre for AgriBioscience, 5 Ring Road, Bundoora, Victoria 3083, Australia; School of Applied Systems Biology (SASB), La Trobe University, Bundoora, Melbourne, Victoria 3086, Australia; Agriculture Victoria Research, AgriBio Centre for AgriBioscience, 5 Ring Road, Bundoora, Victoria 3083, Australia

## Abstract

The Australian Biosecurity Genomic Database (ABGD) is a curated collection of reference viral genome sequences based on the Australian National Notifiable Disease List of Terrestrial Animals. It was created to facilitate the screening of high-throughput sequencing (HTS) data for the potential presence of viruses associated with notifiable disease. The database includes a single verified sequence (the exemplar species sequence, where relevant) for each of the 60 virus species across 21 viral families that are associated with or cause these notifiable diseases, as recognized by the World Organisation for Animal Health. The open-source ABGD on GitHub provides usage guidance documents and is intended to support building a culture in Australian HTS communities that promotes the use of quality-assured, standardized, and verified databases for Australia’s national biosecurity interests. Future expansion of the database will include the addition of more strains or subtypes for highly variable viruses, viruses causing diseases of aquatic animals, and genomes of other types of pathogens associated with notifiable diseases, such as bacteria.

**Database URL**: https://github.com/ausbiopathgenDB/AustralianBiosecurityGenomicDatabase

## Introduction

The World Organisation for Animal Health (WOAH, founded as OIE) maintains a list of notifiable diseases of terrestrial and aquatic animals that are considered to have a serious impact on animal and/or human health [1]. The timely detection and consistent reporting of these pathogens can enable government agencies to pursue the most appropriate action to prevent transboundary spread of critical animal diseases, including zoonoses [2]. The WOAH list is used to inform the Australian National Notifiable Disease List of Terrestrial Animals that determines the priorities of the national biosecurity system, which includes response planning, building diagnostic capability, and performing surveillance activities [3].

High-throughput sequencing (HTS), also called next-generation sequencing (NGS), is a platform technology that enables the building of smarter, stronger national biosecurity and public health systems for the detection and analysis of novel and existing pathogens, including those associated with notifiable diseases of terrestrial animals for Australia and globally [4–6]. The quality of this powerful capability, however, relies heavily on curated databases of verified genomic sequences, from species and often down to genotype level. In addition, efficient accessibility of data for relevant scientists in governments, academia, and industries, and a willingness to share data, are essential for facilitating comparative analysis of HTS data for accurate identification of pathogens of biosecurity concern.

Currently, the publicly available genomic databases that are commonly used for processing HTS data contain huge volumes of sequences that often encompass all taxa, creating complexities in analysis and impeding rapid pathogen identification. Problematically, these large, non-curated databases also contain many partial and misclassified sequences, leading to incorrect taxonomic identification of pathogens from HTS data [7]. Furthermore, there are existing issues with virus taxonomy and disease aetiology that complicate the usage of these public genomic databases. For example, duck virus hepatitis (DVH) is a notifiable disease in Australia and associated with a variety of viruses with confusing nomenclature. The WOAH Terrestrial Manual [8] states DVH is caused by at least three different genotypes of duck hepatitis A virus (species *Avihepatovirus A*; genus *Avihepatovirus*; family *Picornaviridae*) and two types of duck astrovirus (species *Duck astrovirus*; genus *Avastrovirus*; family *Astroviridae*); however, a recent study suggested that under the existing classification system, DVH has been associated with 10 genera among six viral families [9]. These complexities can lead to ambiguity in genetic lineages of interest and misinterpretation of HTS data, which may unnecessarily impact the efficient and accurate identification of potential biosecurity threats.

There are curated reference sequence databases for host-specific pathogens, such as those causing disease in humans [10], plants [11], and swine [12, 13], and pathogen-specific databases, such as the European Classical Swine Fever Virus Database [14], Bovine Viral Diarrhea Virus Database [15], and African Swine Fever Virus Database [16]. However, no sequence databases exist for pathogens associated with diseases that are notifiable in Australia. Therefore, the Australian Biosecurity Genomic Database (ABGD) for viruses of terrestrial animals has been developed to facilitate screening HTS data generated from environmental or animal samples. The database aims to increase awareness among HTS users about potential risks to Australian animal health and their mandatory biosecurity reporting requirements. Usage of the database in Australian animal disease surveillance and diagnostics will help streamline HTS data processing and improve emergency response systems. The database can also serve as a model for other groups of pathogens associated with notifiable diseases, such as bacteria, and those associated with aquatic and plant diseases, both in Australia and globally.

## Materials and methods

### Database design

The ABGD was established based on the Australian National Notifiable Disease List of Terrestrial Animals [3]. Only viral diseases of terrestrial animal species were included in Version 1 of the database. Notifiable diseases that did not specify the causative agent were further investigated using relevant sources (e.g. WOAH) to determine the viral species to be included in the ABGD. Sequences were acquired from the National Center for Biotechnology Information (NCBI) Nucleotide database, using high-quality whole-genome reference sequences from RefSeq where possible. Each virus species was represented by a single isolate and segmented genomes were merged so that each virus was represented by a single sequence. The taxonomic nomenclature of the ABGD reflects that used by the NCBI RefSeq database at the time of writing.

### Screening for notifiable diseases using the database

The ABGD was tested using six HTS datasets generated from terrestrial animal samples known to contain viruses (Table 1). Four of the datasets were from samples sequenced by Agriculture Victoria Research (AVR) and the remaining two datasets were from studies conducted overseas and available via the Sequence Read Archive, NCBI [17, 18]. Detailed methods describing how the AVR datasets were generated can be found in the Supplementary Data.

**Table 1.** The sequence datasets that were used to evaluate the utility of the Australian Biosecurity Genomic Database in disease investigation

Dataset	Host	Sample	Virus present in sample	Notifiable*	No. of reads	Source
1	Chicken	Cloacal swab	Highly pathogenic avian influenza virus	Yes	5 219 772	AVR
2	Piglet	Ileum	Porcine teschovirus	Yes	143 201 804	AVR
3	Piglet	Jejunum contents	Porcine circovirus 3	No	6 124 328	AVR
4	Lamb	EDTA blood	Border disease virus	No	8 424 068	AVR
5	Pigs	Pooled serum	African swine fever virus	Yes	381 412	PRJNA159635 [17]
6	Cow	Soft palate tissue	Foot-and-mouth disease virus	Yes	23 151 672	PRJNA704976 [18]

* Listed on the Australian National Notifiable Disease List of Terrestrial Animals [3]; AVR: Agriculture Victoria Research.

Four of the datasets contained viruses associated with an animal disease that is notifiable in Australia:

- Dataset 1: A chicken infected with highly pathogenic avian influenza virus (HPAIV) (species *Influenza A virus*; genus *Influenzavirus A*; family *Orthomyxoviridae*), the aetiological agent of the notifiable disease ‘infection with Influenza A viruses in birds’;
- Dataset 2: A piglet infected with porcine teschovirus (PTV) (species *Teschovirus A*; genus *Teschovirus*; family *Picornaviridae*), the aetiological agent of the notifiable disease ‘infection with Teschovirus A (porcine enteroviral encephalomyelitis)’;
- Dataset 5: A pig infected with African swine fever virus (ASFV) (species *African swine fever virus*; genus *Asfivirus*; family *Asfarviridae*), the aetiological agent of the notifiable disease ‘infection with African swine fever virus’;
- Dataset 6: A cow infected with foot-and-mouth disease virus (FMDV) (species *Foot-and-mouth disease virus*; genus *Aphthovirus*; family *Picornaviridae*), the aetiological agent of the notifiable disease ‘infection with foot-and-mouth disease virus’.

The remaining two datasets contained viruses that were genomically related to viruses associated with notifiable disease but belong to a different virus species:

- Dataset 3: A piglet infected with porcine circovirus 3 (PCV-3) (species *Circovirus porcine3*; genus *Circovirus*; family *Circoviridae*), which is in the same genus as porcine circovirus 2 (PCV-2) (species *Circovirus porcine2*), the aetiological agent of the notifiable disease ‘post-weaning multi-systemic wasting syndrome’;
- Dataset 4: A lamb infected with border disease virus (BDV) (species *Pestivirus ovis*; genus *Pestivirus*; family *Flaviviridae*), which is in the same genus as three pestiviruses associated with notifiable diseases:
  - Bovine viral diarrhea virus 2 (BVDV-2) (species *Pestivirus tauri*), the aetiological agent of the notifiable disease ‘infection with bovine viral diarrhea virus (type 2)’;
  - Classical swine fever virus (CSFV) (species *Pestivirus suis*), the aetiological agent of the notifiable disease ‘infection with classical swine fever virus’;
  - Porcine pestivirus (PPeV) (species *Pestivirus australiaense*), the aetiological agent of the notifiable disease ‘infection with Bungowannah virus (porcine myocarditis virus or atypical porcine pestivirus)’.

To screen the six datasets for viruses associated with notifiable disease, the sequence reads were mapped to the ABGD using Burrows–Wheeler aligner-maximum exact matches [19] with default settings. Mapped read counts were attained using the SAMtools flagstat command [20], whereas coverage statistics were calculated using the BBMap pileup command [21]. Geneious [22] was used to visualize the mapped read alignments, determine pairwise nucleotide (nt) identity, produce consensus sequences, and generate phylogenetic trees. Dataset 1 was further analysed using the IRMA: Iterative Refinement Meta-Assembler [23] pipeline to determine the HPAIV subtype.

### Assessment of database performance

**Figure 1.**
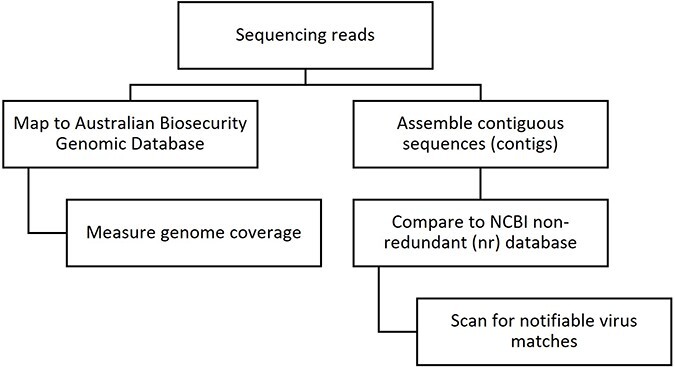
The steps involved in screening HTS data for the presence of viral sequences using database mapping and *de novo* assembly-based approaches.

To assess the performance of the ABGD in disease investigation, the method was compared to a *de novo* assembly-based approach typically used in virome analysis [5]. The database approach involves mapping sequence reads to a targeted database and measuring genome coverage, whereas the assembly approach first assembles the reads into contiguous sequences (contigs) and then compares them to a large non-curated database, such as NCBI’s non-redundant (nr) database (Fig. 1). The assembly approach is popular in virome studies as it enables unbiased detection of all viruses, both known and previously uncharacterized. However, it is a more time- and resource-intensive process that is difficult to interpret and sometimes provides misleading results due to misclassified sequences in public reference databases [7], which can lead to false positives or false negatives in the detection of notifiable viruses.

The datasets containing a notifiable virus (Datasets 1, 2, 5, and 6) were used to measure the performance characteristics of the database and *de novo* assembly-based methods. Contigs were assembled using Trinity [24] with read normalization and trimming options selected, and taxonomically classified using DIAMOND BLASTx [25] with the NCBI nr database (acquired November 2021) and an e-value threshold of 10^–7^. The time taken for both the database screening and *de novo* assembly methods was recorded for each of the four datasets.

## Results and discussion

### Database availability and associated resources

**Figure 2.**
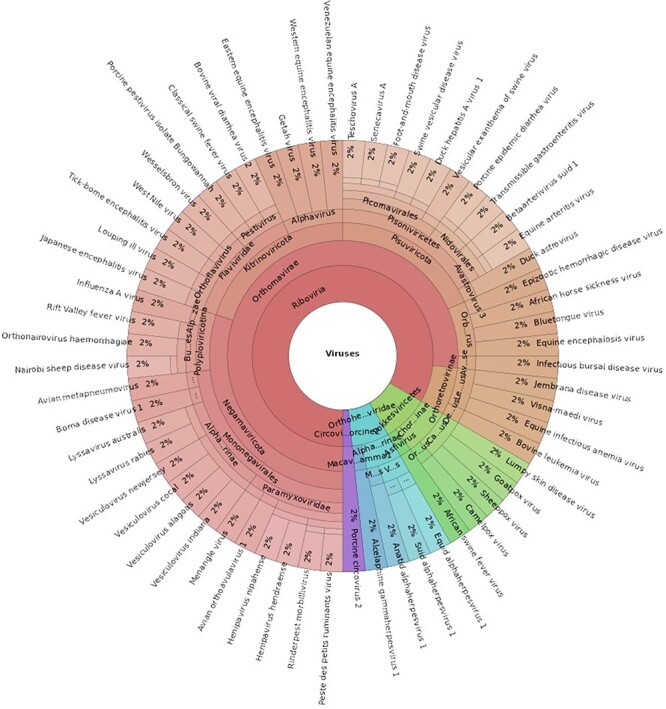
A Krona chart displaying the taxonomical composition of the Australian Biosecurity Genomic Database for viruses of terrestrial animals (an interactive view of this chart can be accessed here: https://htmlview.glitch.me/?https://github.com/ausbiopathgenDB/AustralianBiosecurityGenomicDatabase/blob/main/files/ABGD_taxonomy.krona.html).

The ABGD (Version 1) contains 60 viruses associated with notifiable terrestrial animal diseases in Australia [3], representing 21 viral families, as seen in Fig. 2. The database is provided as a FASTA file that can be used as the reference for read mapping programs and is publicly accessible via GitHub: https://github.com/ausbiopathgenDB/AustralianBiosecurityGenomicDatabase. GitHub facilitates version control of the database by tracking changes to the FASTA file as it is updated and expanded. Users of the database can also submit issues and suggest improvements via the platform and collaborators can be added at any time.

A guide on how to use the database is provided in the README file on the GitHub main page and includes a suggested pipeline for the initial screening of HTS reads, suitable bioinformatic programs, and associated parameters. Proposed thresholds are also provided to help users rapidly interpret the screening results based on initial testing performed in this study, which indicated confidence in results when there was >10% genome coverage by reads with >95% pairwise nt identity, as reported by others [26–30]. Instructions are given on how to follow up any indications of a notifiable virus being present in the sample, including further analyses to collect more data to support the initial result, links to species-specific information, and details of relevant contacts at the Chief Veterinary Office (CVO) for additional assistance.

**Figure 3.**
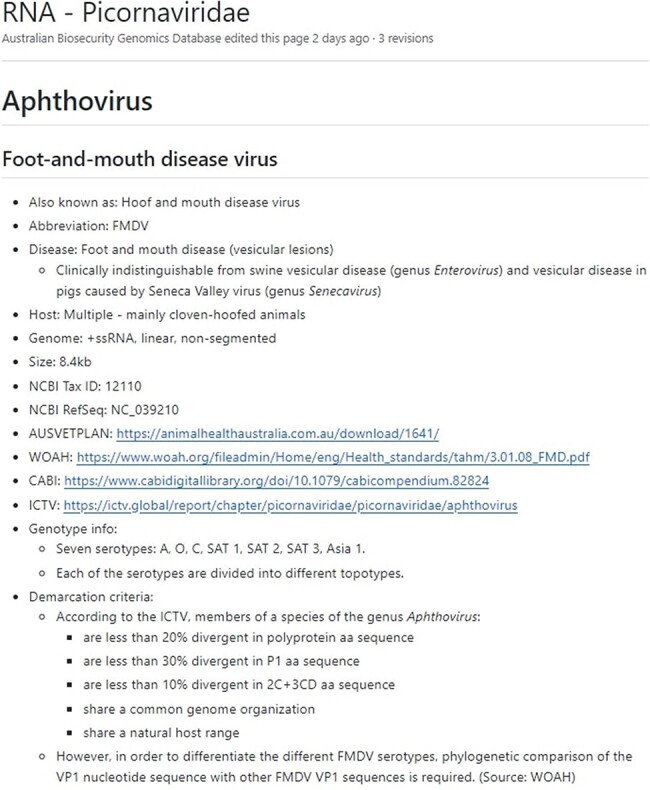
The Notifiable Virus Compendium page for viral species *Foot-and-mouth disease virus*, which belongs to the *Picornaviridae* family and is the aetiological agent of the notifiable foot-and-mouth disease.

Additionally, the ‘wiki’ section of the GitHub (https://github.com/ausbiopathgenDB/AustralianBiosecurityGenomicDatabase/wiki) contains the Notifiable Virus Compendium (NVC), an encyclopaedic resource intended to help a wide variety of users that may not have a diagnostic or animal health background by providing information and resources for each virus species in the database (Fig. 3). The NVC is a useful companion to the database guide, as it provides species-specific information necessary to assess whether the presence of a notifiable virus is indicated in the data via analysis, such as the genome region used for virus species demarcation, and genotypes associated with pathogenicity, thereby reducing ambiguity in interpretation of the Australian National Notifiable Disease List of Terrestrial Animals. Links to diagnostic resources are provided for each species where available, such as WOAH manuals, Australian Veterinary Emergency Plans (AUSVETPLANs), Centre for Agriculture and Biosciences International disease datasheets, and International Committee on Taxonomy of Viruses (ICTV) reports. The taxonomic nomenclature used in the NVC is based on the virus species names accepted by the ICTV at the time of writing. Known nomenclatural synonyms or common names are also listed for each virus species to enable search and access of relevant NVC pages.

### Screening for notifiable diseases

Based on the established usage guidelines, a highly confident result is attained when a genome sequence in the ABGD has >10% coverage by reads with >95% pairwise nt identity. When reads were mapped to the ABGD, sufficient genome coverage and pairwise nt identity were obtained for a highly confident result, indicating the presence of viruses associated with notifiable disease (Table 2). In all instances, the CVO of the local state or territory must be notified. The two datasets containing endemic viruses (Datasets 3 and 4) did not produce >10% genome coverage of the reference sequences in the ABGD.

**Table 2.** A list of the notifiable virus reference sequences that had >10% genome coverage by reads from the sequence datasets and associated coverage metrics

Dataset	Notifiable virus with mapped reads	Mapped reads	% of total reads	Genome coverage (%)	Mean read depth (X)	Pairwise identity (%)
1	Highly pathogenic avian influenza virus	2 612 681	50.05	69.5	25,676.1	99.2
2	Porcine teschovirus	200	<0.01	58.2	3.5	97.5
5	African swine fever virus	1413	0.37	31.4	2.2	94.6
	Rift Valley fever virus	82	0.02	26.8	1.6	98.1
6	Foot-and-mouth disease virus	200	<0.01	17.9	0.7	99.9

Pairwise identity is based on a nucleotide alignment. Visualizations of the genome coverage for each virus can be found in the Supplementary Data.

As outlined in the usage guidelines, read mapping to virus genomes can be used as an initial step to rapidly screen HTS data, but any indication of notifiable virus sequences requires further investigation. This can include other data-based approaches such as inspecting the read mapping alignment to ensure coverage is not being generated by artefacts from the host genome or only present in highly conserved regions that are not specific to species. The alignment can also be used to generate a consensus sequence that can be queried online to check for similarity to other organisms. If present, the data can also be used to determine the genotype or strain of the virus by fulfilling the species demarcation criteria set out by ICTV. This can involve checking the nt or amino acid (aa) identity of a certain gene or performing phylogenetic analysis to see how the sequence groups with others from the virus species.

The detection of a notifiable virus based on HTS results should be followed up by confirmatory testing using an alternative method. The most appropriate tests for each virus can often be found in the relevant WOAH manuals and AUSVETPLANs, which can be accessed via the NVC on the ABGD GitHub. Diagnostic tests can include molecular methods like reverse-transcriptase polymerase chain reaction and Sanger sequencing to confirm the presence of a virus. There are also serological approaches such as virus neutralization, agar gel immunodiffusion, immunofluorescence, passive haemagglutination tests, or enzyme-linked immunosorbent assays. It may also be possible to isolate the virus via cell culture and view it using electron microscopy. The biological characteristics associated with the sample, such as host and symptoms should also be considered, when possible, especially where the sequence information is insufficient to support a more definitive identification.

Each dataset is discussed in detail below to demonstrate how the database screening results can be further investigated to provide support for the initial findings.

### Dataset 1: Highly pathogenic avian influenza virus

The HPAIV genome sequence had 69.5% coverage by 2 612 681 reads from Dataset 1 with 99.2% pairwise nt identity (Table 2). The high number of HPAIV reads in Dataset 1 is reflective of the genome enrichment method used prior to library preparation (see Supplementary Data), compared to the number of reads for each virus target in Datasets 2, 5, and 6, which were sequenced using an untargeted metagenomic approach.

The alignment showed two main areas of the genome lacking coverage (Supplementary Fig. S1A), which corresponded to the two genes coding for the highly variable haemagglutinin (HA) and neuraminidase (NA) surface proteins used to identify AIV subtypes [31]. The HPAIV reference sequence in ABGD originates from a H1N1 isolate, suggesting the HPAIV in Dataset 1 is a different subtype. As mentioned in the NVC, IRMA can be used to characterize highly variable RNA [23] and determined the HPAIV subtype in Dataset 1 to be H7N7, the strain circulating during the 2020 HPAIV outbreak in Victoria, Australia.

These results show that the database can still be used to screen for different HPAIV subtypes with just one reference genome. However, future ABGD expansions should include more genomes for HPAIV subtypes to enable matches across the HA and NA surface proteins. Other notifiable viruses with highly variant subtypes should also be adequately represented in future expansions.

### Dataset 2: Porcine teschovirus

The PTV genome sequence had 58.2% coverage by 200 reads from Dataset 2 with 97.5% pairwise nt identity (Table 2). PTV consists of numerous genotypes of the species *Teschovirus A* and *Teschovirus B* [32], with only *Teschovirus A* being listed as notifiable in Australia [3]. Differentiation of genotypes can be achieved via phylogenetic comparison of the VP1 nt sequence [33]; however, there was no coverage of the VP1 region from the initial database screen (Supplementary Fig. S1B).

Inspection of the *de novo* assembly results revealed a contig containing the VP1 region (uploaded to NCBI: acc. PP996385) and when compared with other PTV VP1 sequences, it grouped with the PTV-17 genotype clade (Supplementary Fig. S2A), with 76.5% nt identity to PTV-17 isolate YC6 (NCBI acc. MN094614). As the ABGD only contains a reference sequence for PTV-1, it is likely that this is responsible for the lack of reads mapping to the VP1 region in the initial screen, reiterating the need to include more subtype reference genomes and highlighting the value of *de novo* assembly as a follow-up analysis.

As explained in the NVC, PTV taxonomy is contentious and often changing, with the ICTV currently recognizing only PTV-1 to -14 as members of *Teschovirus A* [34], whereas a recent study suggests the novel genotypes PTV-17 and -18 are also part of the species [35]. As PTV genotypes can vary in pathogenicity and often do not produce clinical signs [33], biological characteristics such as host and symptoms are helpful in assessing the significance of the finding.

### Dataset 3: Porcine circovirus 3

Despite containing the non-notifiable PCV-3, the Dataset 3 reads did not produce any genome coverage of the notifiable PCV-2. The presence of PCV-3 in Dataset 3 was confirmed by read mapping to a reference PCV-3 genome (NCBI acc. MN075128), producing 76.5% coverage with 99.1% nt identity. The species demarcation criterion for *Circoviridae* is 80% genome-wide pairwise nt identity [36]. This result was supported by phylogenetic analysis of the largest PCV-3 contig (uploaded to NCBI: acc. PP996386) from the *de novo* assembly of the Dataset 3 reads, which grouped with the PCV-3 clade (Supplementary Fig. S2B).

The lack of any PCV-2 genome coverage by the PCV-3 reads in Dataset 3 is most likely due to the high evolutionary rate of the virus [37], demonstrating the specificity of the database when dealing with variable viruses. While PCV-1 and PCV-2 have been previously detected in Australian pig herds [38], the distribution of PCV-3 in Australia is not well defined and its clinical significance is still under investigation [39].

### Dataset 4: Border disease virus

As a member of the *Pestivirus* genus, the non-notifiable BDV in Dataset 4 led to some genome coverage of the notifiable pestiviruses in the ABGD, producing 6.7% coverage of CSFV, 4.7% of BVDV-2, and 0.5% of PPeV, all with 99.8%–99.9% nt identity. Based on usage guidelines provided on the ABGD GitHub, this is a low confidence result with presence of a notifiable virus not well-supported. The species demarcation criteria for the *Pestivirus* genus are based on phylogenetic comparison of conserved aa sequences in several genomic regions [40], which clearly separated the BDV in Dataset 4 from other pestiviruses (Supplementary Fig. S2C). The similarity of the BDV aa sequence (uploaded to NCBI: acc. PP996387) corresponded to the genome coverage results of the initial screen, with 85.1% aa identity to CSFV, 80.3% to BVDV-2, and 70.2% to PPeV. These results demonstrate the utility of the database in providing an indication of non-notifiable viruses, which can still be of use to animal health surveillance.

### Dataset 5: African swine fever virus

The ASFV genome sequence had 31.4% coverage by 1413 reads from Dataset 5 with 94.6% pairwise nt identity (Table 2; Supplementary Fig. S1C). ASFV is the only species within the genus *Asfivirus*, the sole genus in the family *Asfarviridae*; therefore, no nt or aa demarcation criteria have been defined [41]. There are 24 distinct ASFV genotypes [42]; however, all are notifiable in Australia, so no further analysis was performed. In an outbreak situation, phylogenetic comparison of these ASFV sequences with others from previous outbreaks could assist in understanding where the virus originated and how it is being transmitted [43].

In addition to ASFV, Dataset 5 also had 26.8% coverage of the Rift Valley fever virus (RVFV) genome (Table 2), which was not reported in the original 2012 Ugandan study [17]. Sequence reads mapped to all three genome segments (Supplementary Fig. S1D), including a region encoding the RNA-dependent RNA polymerase (RdRp), which when converted to aa sequence, is used for species demarcation [44]. The RVFV RdRp aa reads had ≥95% identity to the RVFV RefSeq genome, satisfying the species demarcation criteria. A 2009 survey of goats in Uganda showed a 9.8% seroprevalence of anti-RVF IgG antibodies, indicating RVFV was circulating in livestock around this time [45]. As there were only 82 reads mapping to 26.8% of the RVFV genome, it is possible these were missed in the original study due to analysis pipeline differences such as stringency of read quality control, alignment parameters, or the reference sequences used.

### Dataset 6: Foot-and-mouth disease virus

The FMDV genome sequence had 17.9% coverage by 200 reads from Dataset 6 with 99.9% nt identity (Table 2). Phylogenetic comparison of the VP1 nt sequence is required to determine the FMDV serotype [46], which showed that the FMDV in Dataset 6 belonged to the Type O serotype (Supplementary Fig. S2D). This corresponds to the FMDV serotype that was used to experimentally infect cattle in the study that generated the dataset reads [18]. As the focus of this study was transcriptional changes in cattle genes in response to FMDV infection, the samples were enriched for mRNA prior to sequencing, which may explain the low number of FMDV reads and genome coverage (Supplementary Fig. S1E). FMDV is one of four species in the genus *Aphthovirus*. The related species *Bovine rhinitis A virus, Bovine rhinitis B virus*, and *Equine rhinitis A virus* are present in Australia [47, 48], and therefore it is important to differentiate species using relevant bioinformatic or laboratory diagnostic techniques.

### Database performance

Mapping reads to the ABGD indicated the presence of sequences belonging to all viruses associated with a notifiable disease in under an hour (Table 3). The *de novo* assembly-based method required at least 86 min to indicate the presence of a notifiable virus sequence, with Dataset 6 requiring almost 10 h of analysis time. Furthermore, assembly of Dataset 6 reads did not produce any FMDV contigs, despite 17.9% of the FMDV genome receiving read coverage when using the database method. Examination of the FMDV genome alignment revealed a lack of overlapping reads (Supplementary Fig. S1E), which would prevent the formation of FMDV contigs, demonstrating the higher sensitivity of the database method.

**Table 3.** Comparison of Australian Biosecurity Genomic Database and *de novo* assembly-based methods indicating the potential presence of notifiable viruses in sequence datasets, including the time taken and the result produced

		Database method		Assembly method	
Dataset	Notifiable virus	Time (min)	Cov. (%)	Time (min)	Contigs
1	Highly pathogenic avian influenza virus	12	69.5	86	49/73
2	Porcine teschovirus	49	58.2	254	10/4867
5	African swine fever virus	4	31.4	152	217/2712
6	Foot–and-mouth disease virus	9.5	17.9	562	0/150 793

The database results are shown as the percentage of notifiable virus genome covered by reads, whereas the assembly results are shown as the number of notifiable virus contiguous sequences (contigs) out of the total number of contigs produced.

## Conclusions and future directions

The ABGD (Version 1) has been used to identify viruses from the Australian National Notifiable Disease List of Terrestrial Animals in HTS datasets from known positive samples. Specifically, identification of HPAIV, PTV, ASFV, and FMDV was performed within an hour, which was comparatively faster and more accurate than *de novo* assembly-based methods often used in virome studies. Furthermore, use of the curated database generated easily interpretable results based on read coverage of virus genomes, thereby providing a streamlined first step in the screening of animal samples and triaging significant viral threats to Australian biosecurity.

Key operational documents available via the GitHub platform underpin quality use of the database. The NVC in the GitHub ‘wiki’ section provides species-specific information necessary to accurately analyse the data, thereby reducing ambiguity in interpretation of the Australian National Notifiable Disease List of Terrestrial Animals. The usage guidelines propose criteria to rapidly assess confidence level (low, medium, and high) in the initial screening results, including thresholds for genome coverage and nt identity. Further testing of the database is recommended to better inform the metrics and thresholds chosen for the screening analysis criteria, which will help to address some of the limitations associated with this approach (Table 4). This should include trialling different read mapping programs and settings with a greater number and variety of datasets, such as those generated by long-read sequencing technology, and from a broad range of sample types and host animals.

**Table 4.** Recommendations to address the possible issues associated with the use of read mapping to a database to screen HTS data for viral sequences

Issue with initial potential detection	Recommended action
Coverage of a notifiable virus genome during the initial screening of the data is due to the presence of a closely related, non-notifiable virus.	Re-analyse sample HTS data to determine if the associated ICTV species/strain demarcation criteria are met.* This may involve generating a consensus sequence and comparing it to the reference to measure the nt or aa similarity of certain genes or genome regions. Read mapping to reference sequences of closely related viral species/strains could also confirm whether the initial screening results are due to coverage of a conserved genome region of a non-notifiable virus.
Coverage of a notifiable virus genome during the initial screening of the data is due to sequencing of the host animal or the presence of a non-viral contaminant.	Re-analyse sample HTS data to determine if the associated ICTV species/strain demarcation criteria are met.* Read mapping to the host animal genome could help determine the specificity of the initial screening results. Alternatively, the virus reads or consensus sequence could be analysed using NCBI’s online BLAST tools to check whether they match the host animal or a non-viral contaminant reference sequence.
Low coverage across the notifiable virus genome or taxonomically informative gene region due to low sequencing depth.	Re-sequence the sample to acquire greater read depth and increase coverage of the virus genome. Alternatively, PCR enrichment of the virus genome or gene regions and Sanger or long-read sequencing of the products could help determine whether the virus of interest is present.
Low coverage across the notifiable virus genome or taxonomically informative gene region due to high variability of the virus.	Re-map the reads to other strains of the notifiable virus to produce greater genome coverage. Expansion of the database to include more than one strain of variable notifiable viruses would also help to address this issue.
A suspected positive detection is based on database read mapping results that do not satisfy the coverage thresholds of the initial screening criteria.	Use other methods to test the sample for the notifiable virus, such as molecular or serology techniques used for laboratory diagnostics. Further development of the specificity and sensitivity criteria used to analyse the database screening results across a range of notifiable diseases, animal species, and sample type would also help to ensure the coverage thresholds used are appropriate.

* If the demarcation criteria cannot be met, the detection may be limited to a genus level. Re-sequencing of the sample to acquire more data could help provide species-level resolution. Further laboratory testing of the sample could also be used to confirm the virus species/strain, including serology, culturing, or PCR methods.

The addition of biological characteristics such as host and symptoms could improve the applicability of the criteria when dealing with low or medium confidence results. However, without clinical or epidemiological data, it is difficult to interpret reporting requirements based only on the detection of nucleic acid, as this does not necessarily indicate an active infection or disease state. There is a need for policymakers to develop clear guidelines for interpreting and reporting HTS-based detections of pathogens in the absence of epidemiological context, which should delineate when genomic findings warrant notification and what follow-up actions are required [49]. More detailed species demarcation criteria for certain genera and advancements in automated taxonomic classification using only sequence data will also help improve the interpretation of results using the ABGD in the future [50].

There is national awareness of the database through the Australian Subcommittee on Animal Health Laboratory Standards [51] and Laboratories for Emergency Animal Disease Diagnosis and Response network [52], and among HTS users by way of scientific forums. The usage of the database will assist in the application of HTS to animal disease surveillance and diagnostics, thereby strengthening Australia’s biosecurity preparedness. The database could also be used in the creation of standardized HTS data processing and interpretation frameworks, to enable harmonized response networks and comparison of surveillance systems at a national level. Furthermore, a database curated to Australian biosecurity priorities can be used in training modules for researchers and laboratory workers not familiar with biosurveillance and emergency response, helping to bridge knowledge gaps and raise awareness of reporting obligations.

Increased awareness of the database could be achieved with the creation of a stand-alone website explaining the aim of the project, collaborating institutions, and links to the GitHub repository and associated resources, such as HTS diagnostic standards and guidelines. Working with relevant stakeholders to add a link to the website on relevant Australian national and state government websites that list notifiable diseases would also help increase awareness and uptake of the database. Development of a step-by-step eLearning module on both the analysis and reporting of notifiable pathogens would facilitate correct usage of the database by HTS users with a diverse range of experience.

Future expansion of the database should include addition of more strains or subtypes for highly variable viruses, contemporary isolates for viruses with outdated reference genomes, viruses on Australian state/territory notifiable animal disease lists, viruses causing diseases of aquatic animals, and genomes of other types of pathogens associated with notifiable diseases, such as bacteria. Establishing a sequence quality assessment system would help ensure the reference genomes included in the database are reliable, particularly if sequences outside the NCBI RefSeq database are used. This development work is in line with relevant recommendations from the Australian National Biosecurity Committee [53].

## Supplementary Material

baae084_Supp

## Data Availability

The database and associated resources, including usage guidelines and the NVC, are available via GitHub at https://github.com/ausbiopathgenDB/AustralianBiosecurityGenomicDatabase. An interactive chart displaying the taxonomical composition of the database can be accessed at https://htmlview.glitch.me/?https://github.com/ausbiopathgenDB/AustralianBiosecurityGenomicDatabase/blob/main/files/ABGD_taxonomy.krona.html. The PTV, PCV3 and BDV sequences generated from the datasets for phylogenetic analysis have been deposited in GenBank (PP996385–PP996387).
