# Supplementary material for: The Australian Biosecurity Genomic Database: a new resource for high-throughput sequencing analysis based on the National Notifiable Disease List of Terrestrial Animals
Source: Database (Oxford). 2024 Aug 28;2024:baae084. doi: 10.1093/database/baae084 (PMC11352597; doi:10.1093/database/baae084)

# Supplementary Data

# Methods used to generate AVR datasets

Dataset 1: Highly pathogenic avian influenza virus

Nucleic acid was extracted from the cloacal swab sample using a MagMAX-96 Viral RNA Isolation Kit (Applied Biosystems) according to manufacturer’s protocol. After screening for avian influenza (AIV) using an Influenza A matrix TaqMan qPCR assay (Heine and Trinidad, 2006), whole genome amplification was performed using a SuperScript III One-Step RT-PCR System with Platinum Taq DNA Polymerase (Invitrogen) and universal AIV gene primers (Zhou *et al*. 2009). The amplified sample was fragmented using NEBNext dsDNA Fragmentase (New England Biolabs) and cleaned up using an ISOLATE II PCR and Gel Kit (Bioline) with a 25 µL elution volume. The fragmented AIV amplicons were quantified with a Qubit dsDNA HS Assay Kit (Invitrogen) and approximately 100 ng was used as input into the adenylation step of the NEXTFLEX Rapid Directional RNA-Seq Kit 2.0 (PerkinElmer). Library preparation was performed according to manufacturer’s protocol, using NEXTFLEX Unique Dual Index Barcodes Set A (PerkinElmer) for indexing. The final library was quantified using a Qubit dsDNA HS Assay Kit (Invitrogen) and a High Sensitivity D1000 ScreenTape (Agilent), then sequenced on an Illumina NovaSeq 6000 using 150 bp reads.

Datasets 2 & 3: Porcine teschovirus and porcine circovirus 3

Approximately 30 mg of tissue was dissected from the piglet samples and placed in 600 µL of Buffer RLT (Qiagen) with two glass beads. The samples were homogenised for 3 mins at 30 Hz, rotated, and homogenised again using a TissueLyser II (Qiagen). The homogenised samples were centrifuged for 3 mins at 14,000 rpm and 500 µL of supernatant was removed for nucleic acid extraction. Extraction was performed using a RNeasy Mini Kit (Qiagen) according to manufacturer’s protocol. Samples were eluted in 30 µL of RNase-free water and treated with TURBO DNase (Invitrogen) according to manufacturer’s protocol. After checking sample concentration with a Qubit RNA HS Assay kit (Invitrogen), libraries were prepared for sequencing with a NEXTFLEX Rapid Directional RNA-Seq Kit 2.0 (PerkinElmer) according to manufacturer’s protocol, using NEXTFLEX Unique Dual Index Barcodes Set A (PerkinElmer) for indexing. Quantification of the final libraries was performed using a Qubit dsDNA HS Assay Kit (Invitrogen) and a High Sensitivity D1000 ScreenTape (Agilent). Libraries were pooled at equimolar concentration and sequenced on an Illumina NovaSeq 6000 using 150 bp reads.

Dataset 4: Border disease virus

Nucleic acid was extracted from a lamb EDTA blood sample using a RNeasy Mini Kit (Qiagen) according to manufacturer’s protocol. The sample was eluted in 60 µL of RNase-free water and quantified using a Qubit RNA HS Assay kit (Invitrogen). Library preparation was performed using a NEXTFLEX Rapid Directional RNA-Seq Kit 2.0 (PerkinElmer) according to manufacturer’s protocol, using NEXTFLEX Unique Dual Index Barcodes Set A (PerkinElmer) for indexing. The final library was quantified using a Qubit dsDNA HS Assay Kit (Invitrogen) and a High Sensitivity D1000 ScreenTape (Agilent), then sequenced on an Illumina MiSeq using 150 bp reads.

# Supplementary references

Heine, H., and Trinidad, L. (2006) *Rapid identification and pathotyping of virulent IBDV, NDV and AIV isolates. RIRDC Publication No CSA-24J*. Available at: https://www.australianeggs.org.au/assets/dms-documents/CSA-24JA-Final-Report.pdf (accessed 15 December 2021).

Zhou, B., Donnelly, M. E., Scholes, D. T., St. George, K., Hatta, M., Kawaoka, Y., and Wentworth, D. E. (2009) Single-reaction genomic amplification accelerates sequencing and vaccine production for classical and swine origin human influenza A viruses. *J Virol* 83 (19): 10309–10313.

# Figure S1: Visualisations of the read mapping alignments for each of the notifiable virus genomes with >10% coverage. The short black bars represent mapped reads, whereas the blue curves represent depth of coverage. The numbers on the top indicate the genome length and on the left the read depth.

A) Highly pathogenic avian influenza virus – 69.5% coverage


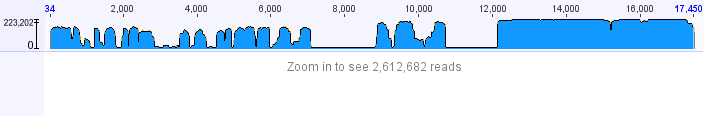


B) Porcine teschovirus – 58.2% coverage


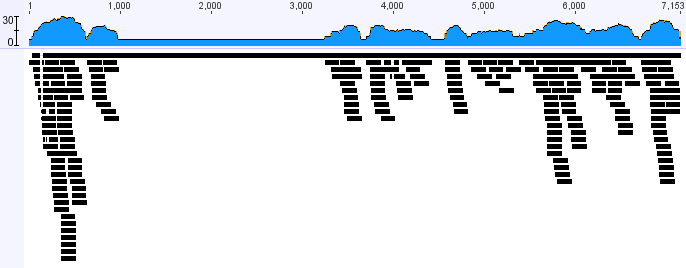


C) African swine fever virus – 31.4% coverage


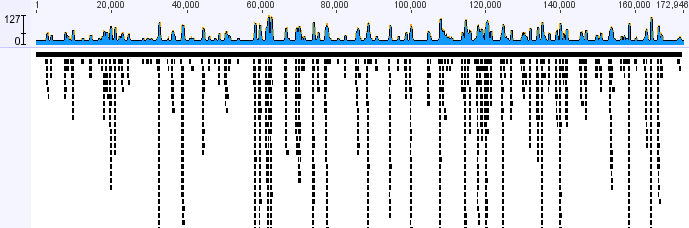


D) Rift Valley fever virus – 26.8% coverage

_
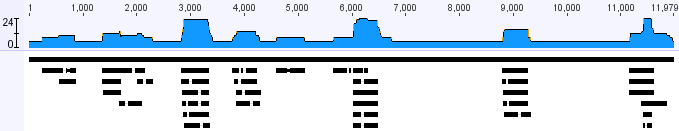
_

E) Foot-and-mouth disease virus – 17.9% coverage


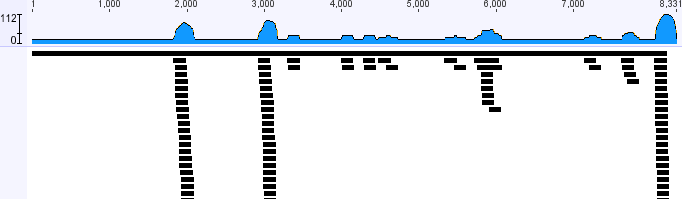


# Figure S2: Phylogenetic trees generated for datasets in Table 1 that required phylogenetic analysis to ascertain the virus species and/or genotype.

A) Phylogenetic tree of a 786 bp region of the VP1 gene of porcine teschoviruses. The Dataset 2 sample contiguous (contig) sequence acquired from assembly is highlighted in red.


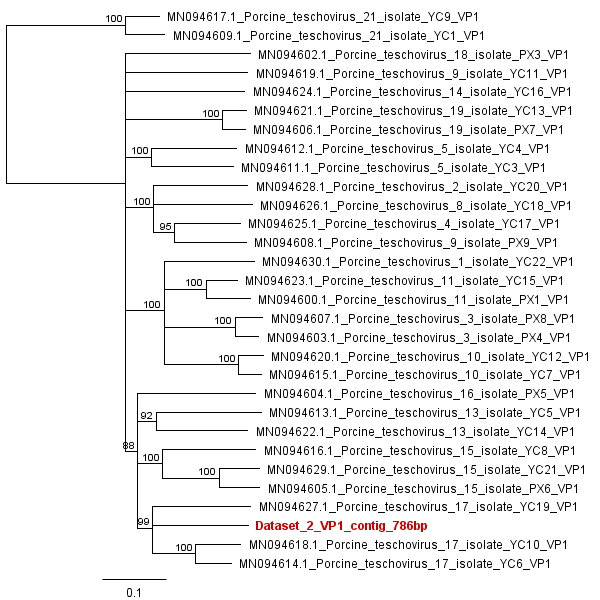


B) Phylogenetic tree of a 559 bp region of porcine circoviruses. Porcine circovirus 3 is coloured in blue, with the Dataset 3 sample contiguous (contig) sequence acquired from assembly highlighted in bold. The notifiable porcine circovirus 2 is coloured in red.


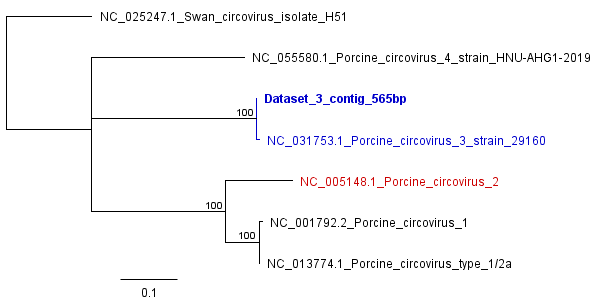


C) Phylogenetic tree of aa positions 3,312–3,899 (numbered according to the first aa of the polyprotein of BVDV-1_M96751) for different species of the *Pestivirus* genus. Border disease virus (BDV) is coloured in blue, with the Dataset 4 sample sequence highlighted in bold. The notifiable bovine viral diarrhea virus 2 (BVDV-2), classical swine fever virus (CSFV), and Porcine pestivirus isolate Bungowannah are coloured in red.


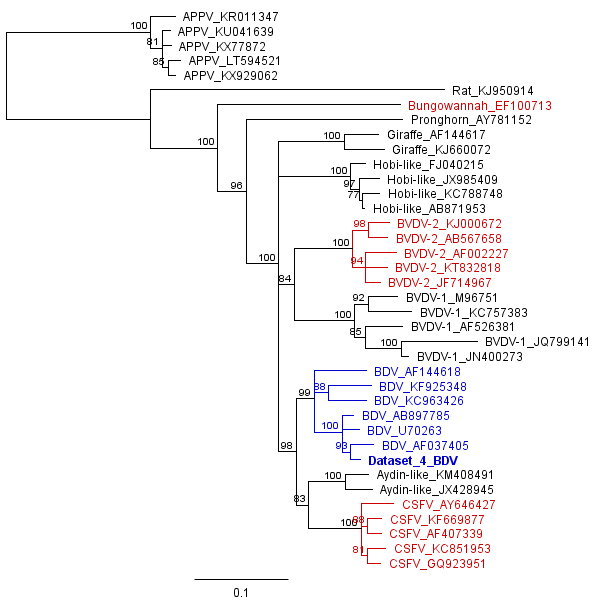


D) Phylogenetic tree of a 155 bp region of the VP1 gene for foot-and-mouth disease virus. The Dataset 6 sample sequence is highlighted in red.


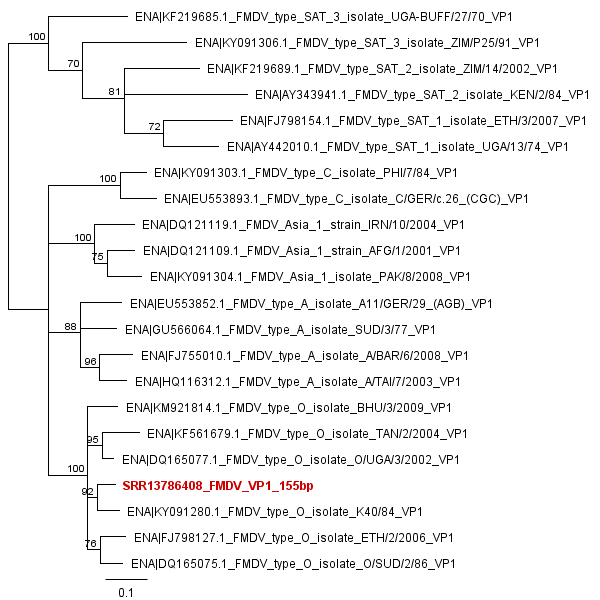

Supplement: baae084_Supp [file baae084_supp.zip › suppl_data/Supplementary data.docx]
